# Occurrence and Ear Damage of *Helicoverpa zea* on Transgenic *Bacillus thuringiensis* Maize in the Field in Texas, U.S. and Its Susceptibility to Vip3A Protein

**DOI:** 10.3390/toxins11020102

**Published:** 2019-02-09

**Authors:** Fei Yang, José C. Santiago González, Jayme Williams, Donald C. Cook, Ryan T. Gilreath, David L. Kerns

**Affiliations:** 1 Department of Entomology, Texas A&M University, College Station, TX 77843-2475, USA; josec.santiago@tamu.edu (J.C.S.G.); rtg006@tamu.edu (R.T.G.); 2 BASF Corporation, Morrisville, NC 27560, USA; jayme.williams@agro.basfse.com; 3 Delta Research and Extension Center, Mississippi State University, Stoneville, MS 38776, USA; dcook@drec.msstate.edu

**Keywords:** Transgenic crops, *Bacillus thuringiensis*, *Helicoverpa zea*, Resistance, Vip3A, Bt Maize

## Abstract

The corn earworm, *Helicoverpa zea* (Boddie), is a major pest of *Bacillus thuringiensis* (Bt) maize and cotton in the U.S. Reduced efficacy of Bt plants expressing Cry1 and Cry2 against *H. zea* has been reported in some areas of the U.S. In this study, we evaluated the occurrence and ear damage of *H. zea* on transgenic Bt maize expressing Cry proteins or a combination of Vip3A and Cry proteins in the field in Texas in 2018. We found that the occurrence of *H. zea* larvae and the viable kernel damage area on the ear were not different between non-Bt maize and Bt maize expressing Cry1A.105+Cry2Ab2 and Cry1Ab+Cry1F proteins. A total of 67.5% of the pyramided Bt maize expressing Cry1Ab+Cry1F+Vip3A was damaged by 2nd–4th instar larvae of *H. zea*. Diet bioassays showed that the resistance ratio against Vip3Aa51 for *H. zea* obtained from Cry1Ab+Cry1F+Vip3A maize was 20.4 compared to a field population collected from Cry1F+Cry1A.105+Cry2Ab2 maize. Leaf tissue bioassays showed that 7-day survivorship on WideStrike3 (Cry1F+Cry1Ac+Vip3A) cotton leaves was significantly higher for the *H. zea* population collected from Cry1Ab+Cry1F+Vip3A maize than for a Bt-susceptible laboratory population. The results generated from this study suggest that *H. zea* has evolved practical resistance to Cry1 and Cry2 proteins. Therefore, it is crucial to ensure the sustainable use of the Vip3A technology in Bt maize and cotton.

## 1. Introduction

Genetically engineered crops expressing *Bacillus thuringiensis* (Bt) proteins have been commercially planted for the control of maize, cotton, and soybean insect pests for more than two decades [1]. In 2017, global adoption of Bt crops reached over 100 million hectares [1]. Besides their high efficacy in controlling target insect pests, these Bt crops also offer environmental and economic benefits, such as reduced chemical insecticide use and crop yield loss [2,3,4,5,6,7]. The widespread use of Bt technology places consistently strong selection pressure on target insect pest populations. Evolution of resistance has become a primary threat to the durability of the Bt crops. From 1996 to 2005, field-evolved practical resistance to Bt crops was only reported in three cases worldwide [8]. However, the cumulative number of cases of field-evolved practical resistance to transgenic Bt crops reached 19 by 2018 [8,9,10,11].

Gene pyramiding, which enables transgenic plants to express two or more dissimilar Bt proteins against the same insect pest, is one of the major strategies currently adopted for insect resistance management in the U.S. [12] Relative to the single Bt protein crops, pyramided Bt crops are expected to be more effective in delaying the evolution of resistance, because when one Bt protein in the pyramids is ineffective, the remaining Bt proteins can kill the insects [13]. Currently, almost all Bt maize hybrids and cotton varieties on the U.S. market are pyramids [14]. The proteins that target the lepidopteran pest in Bt cotton include Cry1Ac, Cry1Ab, Cry1F, Cry2Ab, Cry2Ae, and Vip3Aa19; and the proteins adopted in Bt maize contain Cry1Ab, Cry1A.105, Cry1F, Cry2Ab2, and Vip3Aa20. The very same or similar Bt proteins expressed in these two crops place strong selection pressure on the target insect pests that feed on both crops in areas where maize and cotton are produced in the same landscape. For example, the corn earworm, also called the cotton bollworm, *Helicoverpa zea* (Boddie), is one of the most costly crop insect pests in North America [14,15]. *H. zea* is a major target pest of pyramided Bt maize and Bt cotton in the U.S. Therefore, there is a high potential for multiple generations of *H. zea* exposure to different Bt crops. In addition, studies have indicated that *H. zea* has inherently low susceptibility to Cry1 and Cry2 proteins [16,17,18,19]. All of these factors could create favorable conditions for the fast evolution of resistance to Bt proteins in *H. zea*.

In recent years, *H. zea* control problems in fields of pyramided Bt maize and Bt cotton expressing Cry1/Cry2 have been reported in some areas of the U.S. [20,21,22,23]. For example, Dively et al. [20] documented the field-evolved resistance of *H. zea* to Cry1Ab and Cry1A.105+Cry2Ab2 maize in Maryland. Reisig et al. [24] observed field-evolved practical resistance of *H. zea* to pyramided Bt cotton expressing Cry1Ac+Cry1F and Cry1Ac+Cry2Ab in North Carolina. Diet bioassays conducted by Yang et al. [21,22] also confirmed high resistance ratios for Cry1Ac and Cry2Ab2 purified proteins of *H. zea* populations collected from Louisiana, Mississippi, Arkansas, and Tennessee. Moreover, Cry1/Cry2 proteins are currently used in combination with Vip3A proteins in almost all recently released Bt maize and Bt cotton products in the U.S. Previous studies have shown that there is no or very weak cross-resistance between Vip3A and Cry proteins [25,26,27,28,29,30]. For example, Mahon et al. found that Vip3A-resistant populations of *Helicoverpa armigera* and *Helicoverpa punctigera* have no cross-resistance to Cry1Ac and Cry2Ab proteins [25]. Wei et al. showed that *H. armigera*, with high levels of resistance to Cry1Ac or Cry2Ab, has no cross-resistance to Vip3A [26]. In addition, studies have indicated that cross-resistance is absent among Cry1, Cry2, and Vip3A proteins in Cry1F-resistant, Cry2Ab2-resistant, and Vip3A-resistant populations of *Spodoptera frugiperda* [28,29,30]. Because populations of *H. zea* in most U.S. fields are already resistant to Cry1 and Cry2 proteins, this makes Vip3A the only protein in commercialized Bt crops that is consistently effective against *H. zea*, which markedly raises the risk of resistance [8]. In this study, we reported the occurrence and ear damage of *H. zea* on transgenic Bt maize expressing Cry proteins or a combination of Vip3A and Cry proteins in the field in Texas, U.S. and its susceptibility to Vip3A protein.

## 2. Results

### 2.1. Plant Injury and Occurrence of H. zea on Different Hybrids of Non-Bt and Bt Maize in the Field

The natural occurrence of *H. zea* was high in the field during the experimental period (Table 1). The effects of the treatment on percentage of plants with live larvae, number of larvae per ear, larval development, percentage of plants with damaged ears, and ear damage area were all significant. For the two non-Bt maize hybrids, the mean percentage of plants with live larvae was 76.3%, and the mean number of live larvae per ear was 0.95 (Table 1). Among Cry1Ab+Cry1F and Cry1A.105+Cry2Ab2 maize plants, 91.3% and 83.8%, respectively, were found with live larvae of *H. zea*, which was not significantly (*p* > 0.05) different from that of non-Bt plants (Table 1). An average of 1.85 and 1.30 larvae per ear was observed on the primary ears of Cry1Ab+Cry1F and Cry1A.105+Cry2Ab2 maize plants, respectively, which was similar (*p* > 0.05) to that observed on the non-Bt maize. Larval development on Cry1Ab+Cry1F and Cry1A.105+Cry2Ab2 maize plants, as well as the two non-Bt maize plants, were not different (*p* > 0.05) with an average instar of 4.71 (Table 1). All of the non-Bt, Cry1Ab+Cry1F, and Cry1A.105+Cry2Ab2 maize plants suffered severe ear damage, and the damaged area per ear among these four maize hybrids was not different (*p* > 0.05) with an average of 17.0 cm^2^ of viable kernels (Table 1).

**Figure 1:**
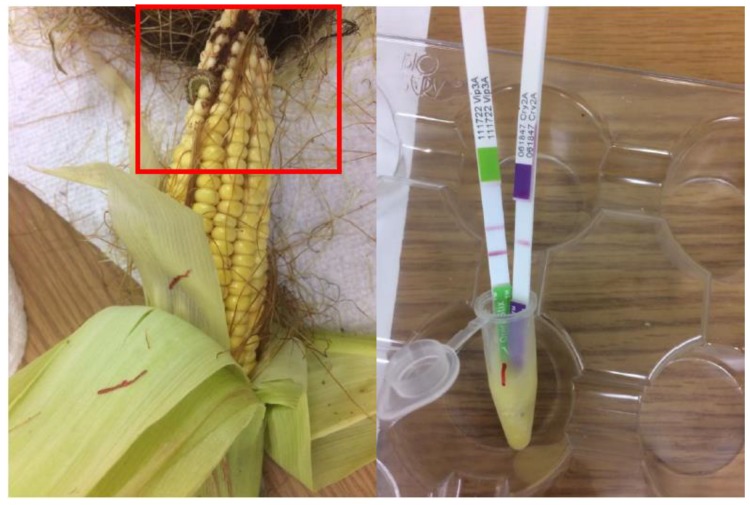
Demonstration of occurrence and ear damage of *Helicoverpa zea* on Leptra maize containing Cry1Ab, Cry1F, and Vip3A proteins; and the Bt protein expression in kernels removed from ears of Leptra maize on QuickStix Combo ELISA test strips (EnviroLogix, ME, USA).

A high infestation of *H. zea* larvae was observed on Bt maize expressing Cry1Ab+Cry1F+Vip3A proteins (Table 1 and Figure 1). In general, 61.3% of Cry1Ab+Cry1F+Vip3A maize was found with an average of 0.79 larvae per ear, which did not differ (*p* > 0.05) from that observed on the non-Bt maize plants (Table 1). Larval development of *H. zea* on Cry1Ab+Cry1F+Vip3A maize ears was significantly (*p* < 0.05) delayed compared to that on non-Bt maize ears. The average larval development index of larvae recovered from non-Bt plants was 4.90, while it was 3.19 for the larvae found on Cry1Ab+Cry1F+Vip3A maize ears (Table 1). In addition, 67.5% of Cry1Ab+Cry1F+Vip3A maize was found with ear damage, and the damaged area per ear was estimated to be 1.3 cm^2^ of viable kernels. These two values were significantly (*p* < 0.05) less than that of the non-Bt maize plants (Table 1).

### 2.2. Susceptibility of Two Different Field Populations of H. zea to Vip3A Protein in Diet Bioassays

Approximately 100 live 2nd to 4th instar larvae recovered from ears of Cry1Ab+Cry1F+Vip3A maize were collected and taken to the laboratory. Simultaneously, ~150 2nd to 5th instar larvae were collected from ears of Cry1F+Cry1A.105+Cry2Ab2 maize plants in another maize field at the same location. F_1_ neonates (<24 h old) of these two populations were tested against purified Vip3Aa51 protein in diet overlay bioassays. A probit analysis showed that the LC_50_ value for *H. zea* populations collected from Cry1F+Cry1A.105+Cry2Ab2 maize was 0.041 μg/cm^2^ (95% confidence limit (CL), 0.035–0.049 μg/cm^2^) (Table 2). The LC_50_ value for *H. zea* populations collected from Cry1Ab+Cry1F+Vip3A maize was 0.838 μg/cm^2^ with a 95% CL of 0.686–0.967 μg/cm^2^ (Table 2). Therefore, the LC_50_ value for Vip3Aa51 protein was significantly higher (*p* < 0.05) for the *H. zea* populations collected from Cry1Ab+Cry1F+Vip3A compared to that obtained from Cry1F+Cry1A.105+Cry2Ab2 maize. The estimated resistance ratio for *H. zea* populations collected from Cry1Ab+Cry1F+Vip3A maize relative to *H. zea* populations collected from Cry1F+Cry1A.105+Cry2Ab2 maize was 20.4 (Table 2).

**Figure 2:**
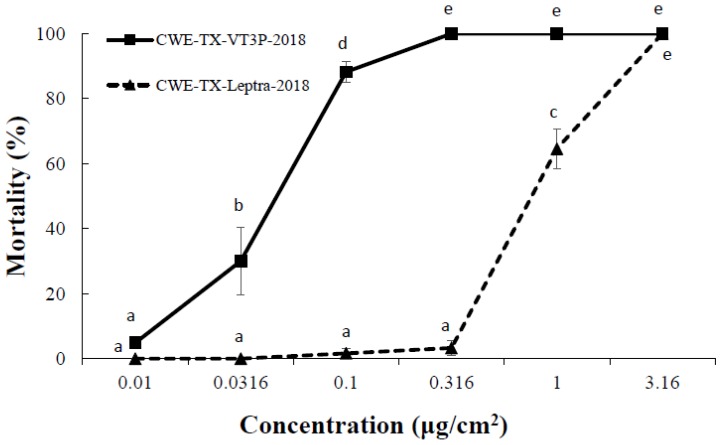
The mortality response of CEW-TX-VT3P-2018 and CEW-TX-Leptra-2018 to Vip3Aa51 protein in diet-overlay bioassays. CEW-TX-VT3P-2018 refers to the *H. zea* population collected from ears of Cry1F+Cry1A.105+Cry2Ab2 maize plants, and CEW-TX-Leptra-2018 refers to the *H. zea* population recovered from ears of Cry1Ab+Cry1F+Vip3A maize. Mean values followed by the same letter are not significantly different at α = 0.05 (Tukey’s HSD test).

**Figure 3:**
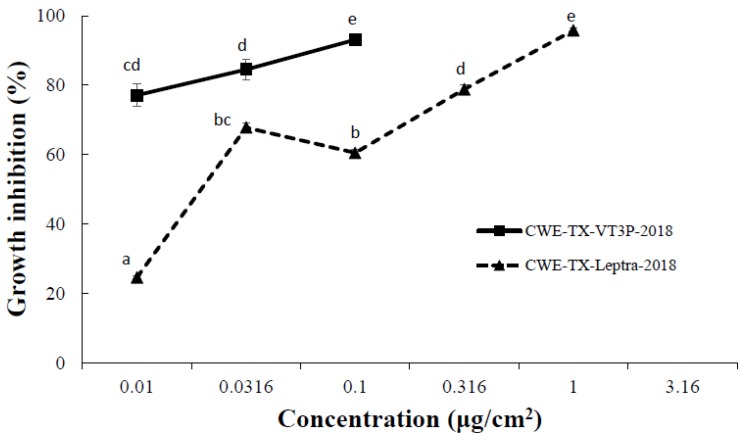
The growth inhibition response of CEW-TX-VT3P-2018 and CEW-TX-Leptra-2018 to Vip3Aa51 protein in diet-overlay bioassays. CEW-TX-VT3P-2018 refers to the *H. zea* population collected from ears of Cry1F+Cry1A.105+Cry2Ab2 maize plants, and CEW-TX-Leptra-2018 refers to the *H. zea* population recovered from ears of Cry1Ab+Cry1F+Vip3A maize. Mean values followed by the same letter are not significantly different at α = 0.05 (Tukey’s HSD test).

ANOVA tests showed that the main effects of insect populations, protein concentrations, and their interactions on larval mortality and growth inhibition were all significant. Mortality for both populations at 0.01 μg/cm^2^ was low and not significantly different (*p* > 0.05). At 3.16 μg/cm^2^, the mortality for both populations was 100% (Figure 2). However, at each tested concentration from 0.0316–1.0 μg/cm^2^, the mortality of *H. zea* populations collected from Cry1Ab+Cry1F+Vip3A was significantly (*p* < 0.05) lower than that of *H. zea* obtained from Cry1F+Cry1A.105+Cry2Ab2 maize (Figure 2). In addition, larval growth inhibition for *H. zea* collected from Cry1Ab+Cry1F+Vip3A was significantly less (*p* < 0.05) than for *H. zea* from Cry1F+Cry1A.105+Cry2Ab2 maize at each tested concentration from 0.01–0.1 μg/cm^2^ (Figure 3).

### 2.3. Larval Survival and Development of H. zea Populations on Cotton Leaf Tissues

The performance of F_1_ neonates (<24 h old) of *H. zea* collected from Cry1Ab+Cry1F+Vip3A maize plants, along with a laboratory susceptible population (SS), were examined on the non-Bt and WideStrike 3 (expressing Cry1Ac, Cry1F, and Vip3A) cotton leaf tissues. Larval survival and development on non-Bt leaf tissues were not different (*p* > 0.05) between the two populations with an average survivorship of 80.4% and an average instar of 3.42 after 7 days (Table 3). Survivorship of SS on leaf tissues of WideStrike 3 cotton was low (3.3%), and the limited survivors were all 2nd instar (Table 3). However, 41.7% of *H. zea* larvae collected from Cry1Ab+Cry1F+Vip3A maize survived on leaf tissues of WideStrike 3, which was significantly higher (*p* < 0.05) than that of SS on WideStrike 3 cotton leaves. In addition, larval development of *H. zea* collected from Cry1Ab+Cry1F+Vip3A maize reached 2.68 instars on leaf tissues of WideStrike 3 after 7 days, which was also significantly greater (*p* < 0.05) than that of SS (Table 3).

## 3. Discussion

In this study, we evaluated the plant injury and occurrence of *H. zea* on different Bt maize hybrids expressing Cry1, Cry2, and Vip3A proteins in the open fields. We found that Bt maize hybrids containing Cry1 and/or Cry2 proteins are not effective for control of *H. zea.* As evidenced by the field data, larval occurrence of *H. zea* was not different on non-Bt maize and Bt maize expressing Cry1Ab+Cry1F and Cry1A.105+Cry2Ab2. All of the Bt maize plants expressing Cry1 and/or Cry2 were severely damaged by *H. zea* larvae, with no differences compared to that of non-Bt maize. In addition, we also investigated the occurrence of *H. zea* on Genuity VT Triple Pro (VT3P) containing Cry1F+Cry1A.105+Cry2Ab2 in two additional independent fields located at the same research farm in 2018 (Table S1). The results also confirmed that Bt maize expressing Cry1F+Cry1A.105+Cry2Ab2 is not efficacious against *H. zea.* Furthermore, diet-overlay bioassays showed that the population of *H. zea* collected from this location exhibited a significant resistance ratio to Cry1Ac (>316-fold) and Cry2Ab2 (24.7-fold) proteins relative to a laboratory susceptible strain (Table S2). The term “practical resistance” has been defined as field-evolved resistance that reduces pesticide efficacy to a level that has consequences for pest control [31]. The data generated from this study clearly demonstrated that *H. zea* has evolved practical resistance to Cry1 and Cry2 proteins in Texas.

Previous studies have shown that *H. zea* has already developed a resistance to the Cry1 and Cry2 proteins used in both Bt maize and cotton plants in some states in the U.S., such as Maryland, North Carolina, South Carolina, Louisiana, Mississippi, Arkansas, and Tennessee [20,21,22,23,24]. In this study, we provide the first report of field control problems of *H. zea* with Cry1 and/or Cry2 Bt maize in Texas. Various factors are speculated to have contributed to the fast and widespread resistance of this insect pest to Cry1/Cry2 proteins in the U.S., especially in the southern regions. For example, a reduced refuge size from 50% to 20% for pyramided Bt maize has been adopted in the southern regions, which will reduce the refuge populations. Cross-pollination and larval movement in seed-mixed plantings can kill susceptible insects but allow heterozygotes to survive, thus increasing the dominance of resistance and accelerating the evolution of resistance [32,33,34]. Additionally, the overlap of Cry1 and Cry2 proteins in Bt maize and cotton could increase the selection pressure for resistance. Finally, the overwintering behavior of *H. zea* in southern regions would allow for resistant alleles to be carried to the next year’s population gene pool and exacerbate the risk of perpetuating resistance [35,36,37]. With the field-evolved resistance of *H. zea* to Cry1 and Cry2 proteins, Vip3A is the only effective protein against *H. zea* in Bt maize and cotton crops, and thus the effectiveness of the pyramids containing Vip3A and Cry1/Cry2 to delay the evolution of resistance will be challenged. Therefore, resistance management must be adopted to ensure the sustainability of the Vip3A technology.

Vip3Aa maize and cotton have been commercially grown in the U.S. since 2010 and 2014, respectively [38,39]. Previous studies have suggested that Bt maize expressing Vip3A protein is highly effective against *H. zea*, even in seed-mixed plantings of Bt and non-Bt maize [40,41]. Field bioassay data also showed that Vip3A technology could provide exceptional protection against *H. zea* damage [24,42,43,44]. High efficacy of Leptra maize expressing Cry1Ab, Cry1F, and Vip3A proteins against *H. zea* has been reported in several studies [44,45]. For example, Reay-Jones et al. indicated that ear injury in Leptra maize, averaged across locations in the southern U.S., ranged from <0.01 cm^2^ per ear in 2014 to 0.05 cm^2^ per ear in 2012 [44]. They also showed that ear damage in 100% Leptra maize plots ranged, across locations, from no injury to 0.42 cm^2^ per ear in Mississippi in 2013 [44]. Bilbo et al. evaluated the efficacy of Leptra maize for control of *H. zea* during 2014–2016 in North Carolina, South Carolina, and Mississippi. They found only very limited viable kernel injury and that the addition of Vip3Aa20 to Cry1Ab+Cry1F greatly increased the toxicity to *H. zea* [45]. In this study, Leptra maize provided exceptional *H. zea* efficacy as reduced viable kernel damage relative to non-Bt and Bt maize expressing Cry proteins. For example, ear injury in Leptra maize was only 1.3 cm^2^ per ear. Based on damaged area per ear, the efficacy of Leptra was 93% relative to the non-Bt hybrids.

In this study, we observed a significant number of *H. zea* larvae on Leptra maize ears expressing Cry1Ab, Cry1F, and Vip3A proteins. Larval occurrence regarding the percentage of plants containing live larvae and the number of larvae per ear on Cry1Ab+Cry1F+Vip3A maize was not different from that on the non-Bt maize plants. Diet-overlay bioassays with Vip3Aa51 protein showed that *H. zea* populations collected from Cry1Ab+Cry1F+Vip3A maize had a significant resistance ratio (>20-fold) relative to a population of *H. zea* collected from Cry1F+Cry1A.105+Cry2Ab2 maize. In addition, compared to the average LC_50_ value (0.09 μg/cm^2^) of Vip3Aa51 from 21 field populations collected in Texas, Mississippi, Louisiana, Arkansas, and Tennessee in 2018 (Yang et al. unpublished data), the *H. zea* populations collected from Cry1Ab+Cry1F+Vip3A maize approximated a 9.3-fold resistance ratio. Furthermore, a cotton leaf bioassay indicated that the larvae collected from Cry1Ab+Cry1F+Vip3A maize could survive well on WideStrike3 leaves containing Cry1Ac+Cry1F+Vip3A proteins. In conclusion, these data, taken together, suggest that the *H. zea* population collected from Cry1Ab+Cry1F+Vip3A maize showed significantly reduced susceptibility against Vip3A proteins. A study has indicated that maize producing Vip3A protein alone does not meet the high-dose criterion for management of *H. zea* [38]. Since Cry1Ab and Cry1F are not highly effective in killing *H. zea* larvae [46] and *H. zea* has developed field resistance to Cry1Ac and Cry1A.105 proteins [20,24], Vip3A is the only effective protein in Leptra maize for the management of *H. zea* populations. It is possible that the low resistance ratio to Vip3Aa51 detected in the protein bioassay could be enough to allow this population to survive on the maize ears. Further research will focus on enhancing the resistance level of this Vip3A-tolerant *H. zea* population and test whether they can survive and develop on ears of maize plants expressing the Vip3A protein.

In conclusion, data generated from this study clearly show that *H. zea* has developed a practical resistance to Bt maize plants expressing Cry1 and Cry2 proteins in Texas. We also detected a high infestation of *H. zea* on Cry1Ab+Cry1F+Vip3A maize in the field. As far as we know, this is the first study to detect a high infestation of *H. zea* on Bt maize expressing Vip3A protein in the field in the U.S. The results generated from this study should provide valuable information for promoting the sustainable use of Vip3A technology for the control of *H. zea* and improving insect resistance management strategies.

## 4. Materials and Methods

### 4.1. Source of Bt and Non-Bt Maize Hybrids

Plant injury and occurrence of *H. zea* in the field were evaluated against five maize hybrids, including two non-Bt and three Bt maize hybrids. The three Bt maize hybrids included DKC 67-72VT2P, Genuity VT Double Pro (VT2P) (Monsanto Company, St. Louis, MO, USA); P1637YHR, Intrasect; and Leptra, P1637VYHR (Pioneer Hi-Bred, Johnson, IA, USA). VT2P contains Cry1A.105 and Cry2Ab2 proteins; Intrasect expresses Cry1Ab and Cry1F proteins; and Leptra contains Cry1Ab, Cry1F, and Vip3A proteins. The two non-Bt maize hybrids used in this study, DKC 67-70RR (Monsanto Company, St. Louis, MO, USA) and P1637R (Pioneer Hi-Bred, Johnson, IA, USA), were genetically closely related to one or two of the three Bt maize hybrids. We randomly sampled one maize ear from the middle two rows in each plot. These ears were brought back to the laboratory and were used for the qualitative ELISA tests. Expression/non-expression of the Cry and Vip3A proteins in the maize hybrids were confirmed using an ELISA-based technique (EnviroLogix, Quantiplate^TM^ kits, Portland, ME, USA). ELISA tests were conducted according to the protocol’s procedure manual (EnviroLogix, Quantiplate^TM^ kits, Portland, ME, USA).

### 4.2. Field Planting

A field trial was conducted on the Texas A&M University Farm in Snook, Texas on 26 March 2018 (34.999490° N; 101.918570° W). Each maize hybrid was planted on 3.1-m wide × 9.1-m long plots. Each plot contained four 0.72-m wide rows, and each row contained about 43 plants. The distance between each plot was approximately 3 feet. These plots were arranged in a randomized complete block design with four plots (blocks) for each maize hybrid. Plant injury and occurrence of natural populations of *H. zea* were closely monitored and checked on the primary ears. In each plot, 20 plants per row were randomly sampled. Percentage of plants with live larvae, number of larvae per ear, larval development, percentage of plants with damaged ears, and area of viable kernel damage were recorded on 22 June 2018.

### 4.3. Dose-Response Bioassays

Susceptibility to Vip3A of two F_1_ populations of *H. zea* was evaluated using a diet-overlay bioassay as described in Anilkumar et al. and Yang et al. [30,47]. The first population was established from approximately 100 live 2nd to 4th instar larvae recovered from ears of Cry1Ab+Cry1F+Vip3A maize in the field on 22 June 2018. The second population consisted of ~150 2nd to 5th instar larvae collected from ears of Cry1F+Cry1A.105+Cry2Ab2 maize plants at the same farm and on the same day as the first population. These field-collected larvae were reared on an artificial diet (WARD’S Stonefly *Heliothis* diet, Rochester, NY, USA) under 26 ± 1 °C, 50% relative humidity (RH), and a 16:8 h (Light:Dark) photoperiod until the pupal stage. The Vip3Aa51 protein was provided by BASF Company (Research Triangle Park, NC, USA) in solution at a concentration of 2.9 mg/mL. The protein was stored in 50 mM Caps, pH 10.5, 10% glycerol, 1 mMDTT, and 10 mM maltose. The sequence information of the Vip3Aa51 protein can be retrieved from the NCBI with the GenBank Accession: KC156649.1. It shows 94.93% homology compared to Vip3Aa19. Each bioassay consisted of seven concentrations ranging from 0 to 3.16 μg/cm^2^. Repeater pipets were used to dispense 0.8 mL per well of liquid diet (Southland Product, Inc., Lake Village, AR, USA) into 128-well bioassay trays (C-D International, Pitman, NJ, USA). Once the diet cooled and solidified, a volume of 40 μL Vip3A protein solution suspended in 0.1% Triton-X100 was overlaid onto the diet surface of each well and allowed to air dry. One neonate (<24 h) was released on the diet surface in each well. After larval inoculation, wells were covered with vented lids (C-D International, Pitman, NJ, USA). Each combination of insect population by Vip3Aa51 protein concentration was replicated four times with 16 larvae in each replication. Bioassay trays were placed in an environmental chamber maintained at 26 ± 1 °C, 50% RH, and a 16:8 (L:D) h photoperiod. Larval mortality and larval weight were recorded on the 7th day after inoculation.

### 4.4. Cotton Leaf Tissue Bioassays

The performance of F_1_ neonates (<24-h old) of *H. zea* collected from Cry1Ab+Cry1F+Vip3A maize plants, along with a laboratory susceptible population (SS) collected at the LSU AgCenter Macon Ridge Research Station in Franklin Parish in May 2016, were examined on the non-Bt, PHY 425RF and WideStrike 3, and PHY 480W3FE (Dow AgroScience, Indianapolis, IN, USA) (expressing Cry1Ac, Cry1F, and Vip3A) cotton leaf tissues. Cotton leaf tissues were collected from field-grown plants on a Texas A&M University Farm in Snook, Texas in 2018. Expression/non-expression of the Cry and Vip3A proteins in the cotton varieties were confirmed using an ELISA-based technique (EnviroLogix, Quantiplate^TM^ kits, Portland, ME, USA). In the leaf tissue bioassays, one leaf of a cotton variety was placed in each well of 8-well trays (C-D International, Pitman, NJ, USA), and five neonates of one of the two *H. zea* populations were then placed on the leaf tissue in the well. In each leaf tissue bioassay, there were four replications for each combination of cotton product and insect population, and each replication consisted of six wells each with five larvae (n = 4 × 30 = 120). Bioassay trays with larvae and leaf tissue were maintained at 26 ± 1 °C, 50% RH, and a 16:8 (L:D) h photoperiod. Leaves were replaced every two days. Larval survival and development were recorded on the 7th day after infestation.

### 4.5. Data Analysis

In the field assay and cotton leaf bioassay, data on the number of larvae per ear, the average instar, and the ear damage area were transformed to the log(x + 1) scale, while the percentage of plants with live larvae, the percentage of plants with damaged ears, and larval survivorship were transformed using the arcsine of (x^0.5^) to normalize treatment variances. The transformed data were then analyzed using one-way or two-way analysis of variance with maize hybrids, insect populations, and cotton varieties as the main factors [48]. Treatment means were separated using Tukey’s honestly significantly different (HSD) test at the α = 0.05 level. Untransformed data are presented in tables.

In the protein bioassay, larval growth inhibition was calculated using the formula: growth inhibition (%) = 100 * (body weight of larvae feeding on the control diet − body weight of larvae feeding on a Vip3Aa51 protein-treated diet)/(body weight of larvae feeding on the control diet). Larval mortality was calculated as mortality (%) = 100 * (number of dead larvae + number of surviving larvae that were still in the first instar)/total number of insects assayed, and the larval mortality value at each concentration was corrected based on the control mortality. A probit analysis was used to determine the median lethal concentration (LC_50_) that caused 50% mortality and the corresponding 95% confidence limit (CL) [48]. Resistance ratio was calculated using the LC_50_ of the *H. zea* population collected from Cry1Ab+Cry1F+Vip3A maize, divided by the LC_50_ of the population collected from Cry1F+Cry1A.105+Cry2Ab2 maize. Moreover, larval mortality and growth inhibition were analyzed using a two-way ANOVA with insect population and protein concentration as the two main factors [48]. Original data on the percentage of larval mortality and growth inhibition were transformed using an arcsine (χ^0.5^) to meet normality assumptions. Treatment means were separated using Tukey’s HSD test at the α = 0.05 level [48]. Untransformed data are presented in figures.

## Figures and Tables

**Table 1:** Plant injury and occurrence of *Helicoverpa zea* on different hybrids of non-*Bacillus thuringiensis* (Bt) and Bt maize in the field *.

Variety	Bt Proteins	% Plant with Larvae ^#^	Number of Larvae/Ear	Average Instar	% Plant with Damaged Ear ^§^	Damaged Area per Ear (cm^2^)
DKC-NBt	/	77.5 ± 6.0 ab	0.96 ± 0.11 ab	4.86 ± 0.13 bc	100.0 ± 0.0 b	18.1 ± 0.9 b
P-NBt	/	75.0 ± 2.0 ab	0.93 ± 0.05 ab	4.94 ± 0.11 c	100.0 ± 0.0 b	19.1 ± 1.7 b
Intrasect	Cry1Ab+Cry1F	91.3 ± 4.3 b	1.85 ± 0.13 b	4.31 ± 0.06 b	100.0 ± 0.0 b	15.7 ± 1.5 b
VT2P	Cry1A.105+Cry2Ab2	83.8 ± 3.8 ab	1.30 ± 0.18 b	4.71 ± 0.11 bc	100.0 ± 0.0 b	15.0 ± 1.2 b
Leptra	Cry1Ab+Cry1F+Vip3A	61.3 ± 3.1 a	0.79 ± 0.04 a	3.19 ± 0.19 a	67.5 ± 1.4 a	1.3 ± 0.2 a
F-test	*F-value*	*F_4,12_* = 6.16	*F_4,12_* = 13.30	*F_4,12_* = 40.36	*F_4,12_* = 1546.47	*F_4,12_* = 224.56
	*P-value*	0.0062	0.0002	<0.0001	<0.0001	<0.0001

* Mean values within a column followed by the same letter are not significantly different at α = 0.05 (Tukey’s honestly significant difference (HSD) test). ^#^ Percentage of plants with live larvae of *H. zea*. ^§^ Percentage of plants with damaged ears by larvae of *H. zea*.

**Table 2:** The mortality response (LC_50_) of different populations of *Helicoverpa zea* to Vip3Aa51 protein in diet-overlay bioassays.

Insect Population *	N ^#^	LC_50_ (95% CI) (μg/cm^2^) ^§^	Slope ± SE	X^2^	df	Resistance Ratio ^£^
CEW-TX-VT3P-2018	448	0.041 (0.035, 0.050)	2.87 ± 0.30	18.9	22	1.0
CEW-TX-Leptra-2018	448	0.838 (0.686, 0.966)	4.93 ± 1.02	19.0	22	20.4

* CEW-TX-VT3P-2018 refers to the *H. zea* population collected from ears of Cry1F+Cry1A.105+Cry2Ab2 maize plants, and CEW-TX-Leptra-2018 refers to the *H. zea* population recovered from ears of Cry1Ab+Cry1F+Vip3A maize. ^#^ Total number of neonates assayed. ^§^ Median lethal concentration (LC_50_) that caused 50% mortality and the corresponding 95% confidence limit (CL). Larval mortality was calculated based on the number of dead larvae plus survivors that were still in the first instar divided by the total number of insects assayed. ^£^ Resistance ratio was calculated using the LC_50_ value of CEW-TX-Leptra-2018 divided by the LC_50_ of CEW-TX-VT3P-2018. SE, standard error.

**Table 3:** The performance of two different populations of *Helicoverpa zea* on cotton leaf tissues *.

Cotton Variety		Insect ^§^	Survivorship (%) ^£^	Average Instar
Non-Bt		CEW-TX-Leptra-2018	78.3 ± 2.9 c	3.39 ± 0.01 c
		CEW-TX-SS	82.5 ± 3.2 c	3.45 ± 0.05 c
WideStrike 3		CEW-TX-Leptra-2018	41.7 ± 7.5 b	2.68 ± 0.03 b
		CEW-TX-SS	3.3 ± 1.4 a	2.00 ± 0.00 a
F-test	Insect	F-value	*F*_1, 12_ = 19.83	*F*_1, 11_ = 161.46
		*p*-value	0.0008	<0.0001
	Cotton variety	F-value	*F*_1, 12_ = 158.41	*F*_1, 11_ = 1483.01
		*p*-value	<0.0001	< 0.0001
	Insect * Cotton variety	F-value	*F*_1, 12_ = 29.64	*F*_1, 11_ = 215.14
		*p*-value	<0.0001	<0.0001

* Mean values within a column followed by the same letter are not significantly different at α = 0.05 (Tukey’s HSD test). ^§^ CEW-TX-Leptra-2018 refers to the *H. zea* population recovered from ears of Cry1Ab+Cry1F+Vip3A maize, and CEW-TX-SS is a laboratory susceptible colony, which has been documented to be susceptible to Cry1Ac, Cry2Ab2, and Vip3A protein. ^£^ Larval survivorship was calculated based on the number of live larvae that were in the second instar and above divided by the total number of insects assayed [28].

## Supplementary Materials

The following are available online at https://www.mdpi.com/2072-6651/11/2/102/s1. Table S1: Occurrence of *Helicoverpa zea* on Genuity VT Triple Pro (VT3P) Bt maize in the fields in 2018. Table S2: LC_50_ and 95% confidence limits (CL) based on larval mortality of *Helicoverpa zea* collected from Genuity VT Double Pro (VT2P) Bt maize in the fields in 2018 to Cry1Ac and Cry2Ab2 proteins.
