# Supplementary material for: Occurrence and Ear Damage of Helicoverpa zea on Transgenic Bacillus thuringiensis Maize in the Field in Texas, U.S. and Its Susceptibility to Vip3A Protein
Source: Toxins (Basel). 2019 Feb 9;11(2):102. doi: 10.3390/toxins11020102 (PMC6416581; doi:10.3390/toxins11020102)
Supplement: Supplementary file 1 [file toxins-11-00102-s001.pdf]

Supplementary Materials: Occurrence and Ear Damage of *Helicoverpa zea* on Transgenic *Bacillus thuringiensis* Maize in the Field in Texas, U.S. and Its Susceptibility to Vip3A Protein

Fei Yang, José C. Santiago González, Jayme Williams, Donald C. Cook, Ryan T. Gilreath, and David L. Kerns

Table S1. Occurrence of *Helicoverpa zea* on Genuity VT Triple Pro (VT3P) Bt maize in the fields in 2018 *.

Trial	No. Larvae Per Ear	Average Instar	% Plant with Larvae #
A	1.59 ± 0.10	3.83 ± 0.07	93.33 ± 0.00
B	1.87 ± 0.13	3.88 ± 0.05	96.67 ± 1.93

* Larval occurrence of natural populations of *H. zea* was checked in two independent fields of Genuity VT Triple Pro (VT3P) containing Cry1F+Cry1A.105+Cry2Ab2 proteins. In each field trial, there were four replications and each replication contained 30 plants. # Percentage of plants with live larvae of *H. zea.* Field trial A and B were conducted on a Texas A&M University Farm in Snook, Texas in 2018 (34.999490° N; 101.918570° W).

Table S2. LC_{50} and 95% confidence limits (CL) based on larval mortality of *Helicoverpa zea* collected from Genuity VT Double Pro (VT2P) Bt maize in the field in 2018 to Cry1Ac and Cry2Ab2 proteins.

Bt Protein [a]	Insect Strain [b]	N [c]	LC_{50} (95% CL) (μg/cm²) [d]	Slope ± SE	X^2	df	Resistance Ratio [e]
Cry1Ac	CEW-BZ-SS	958	0.10 (0.08, 0.11)	1.55 ± 0.09	28.4	26	1.0
	CEW-TX-VT2P	448	> 31.60	/	/	/	> 316.0 *
Cry2Ab2	CEW-BZ-SS	960	0.20 (0.17, 0.24)	1.61 ± 0.09	30.7	26	1.0
	CEW-TX-VT2P	448	4.95 (3.31, 8.40)	1.01 ± 0.11	15.6	22	24.7 *

[a] Cry1Ac protein was provided by Bayer CropScience as lyophilized MVPII powders with 20.0% AI. Cry2Ab2 protein was provided by Bayer CropScience in the form of lyophilized (freeze-dried) Bt-corn leaf powder expressing ~4 mg of Cry2Ab2 protein/g.
[b] CEW-BZ-SS was obtained from a commercial source Benzon Research Inc., Carlisle, PA (CEW-BZ-SS). CEW-TX-VT2P were collected from Genuity VT Double Pro (VT2P) Bt maize on a Texas A&M University Farm in Snook, Texas in 2018 (34.999490° N; 101.918570° W).
[c] Total number of neonates assayed.
[d] The LC_{50} value of an insect strain was considered to be greater than the highest Bt protein concentration used in the bioassay if its larval mortality was <50% at the highest concentration. Larval mortality was calculated based on the number of dead larvae plus survivors that were still in the first instar divided by the total number of insects assayed.
[e] Resistance ratio for a Bt protein was calculated by dividing the LC_{50} value of the *H. zea* population collected from Genuity VT Double Pro (VT2P) Bt maize (CEW-TX-VT2P) by that of the susceptible strain (CEW-BZ-SS).
* indicates significant resistance ratios that were >10-fold.
